# Massive underreporting of type II Diabetes in emergency department admissions

**DOI:** 10.1186/1757-7241-21-S2-A35

**Published:** 2013-09-09

**Authors:** Josefin Gustafsson, Søren Wistisen Rasmussen, Thomas Schmidt-Andersen

**Affiliations:** 1 The Emergency Department, Holbaek University Hospital, Denmark

## Background

The prevalence of type 2 diabetes mellitus (T2DM) has continued to increase in developed and developing countries in past decades. T2DM is the most frequent chronic disease in Denmark with a prevalence of 4 %, with an equal estimated prevalence of undiagnosed cases. T2DM is associated with several complications that can lead to acute deterioration and need of acute admittance to the hospital. Every day 10 patients die from T2DM-related complications in Denmark. The objectives of this study were: 1) to establish the number of patients with T2DM referred to the Department of Internal Medicine via the Emergency Department and the number of patients who were diagnosed with T2DM upon referral; 2) to report which anti-diabetics they used at the time of admittance.

## Methods

A chart review during a 1 year period was conducted, i.e. from 01-JUL-2011 to 30-JUN-2012 looking at all registered cases of T2DM including both primary and secondary diagnosis.

## Results

The Emergency Department yearly admits and refers around 18,000 internal medicine patients. A total of only 31 cases with T2DM were captured. Twenty-eight were known to have T2DM and three were diagnosed with the disease at admittance. Oral anti-diabetics had been prescribed to 67% of patients, oral anti-diabetics in combination with either GLP-1 agonist or insulin to 17%, leaving 13% of the patients with insulin.

## Conclusion

With a T2DM prevalence of 4%, the expected number of admissions was 720 patients. This indicates an underreporting of T2DM of approximately 96%, which probably results from registration of complications related to T2DM and not the underlying disease itself. Because T2DM is regarded as a global epidemic, registration of patients with T2DM needs to be improved so that the quality of both primary and secondary care can be ensured. Because of underreporting the reported anti-diabetic treatment is unlikely to be representative.

